# STIL Promotes Tumorigenesis of Bladder Cancer by Activating PI3K/AKT/mTOR Signaling Pathway and Targeting C-Myc

**DOI:** 10.3390/cancers14235777

**Published:** 2022-11-24

**Authors:** Hua Yu, Liang Chen, Xia Wang, Feng Tang, Ziyu Wan, Hao Wang, Qiqi Fu, Zhizhuang Chen, Jiageng Shi, Xuan Hu, Yisha Zuhaer, Madanyeti Aersi, Tao Liu, Huangheng Tao, Jianping Peng

**Affiliations:** 1 Department of Urology, Zhongnan Hospital of Wuhan University, Wuhan 430061, China; 2 Medical Science Research Center, Zhongnan Hospital of Wuhan University, Wuhan 430061, China; 3 Department of Public Health, Wuhan University Hospital, Wuhan University, Wuhan 430072, China; 4 State Key Laboratory Breeding Base of Basic Science of Stomatology (Hubei-MOST), Wuhan University, Wuhan 430070, China; 5 Key Laboratory for Oral Biomedicine of Ministry of Education (KLOBM), Wuhan University, Wuhan 430070, China; 6 School and Hospital of Stomatology, Wuhan University, Wuhan 430070, China

**Keywords:** STIL, bladder cancer, PI3K/AKT/mTOR pathway, c-myc, cell cycle

## Abstract

**Simple Summary:**

Currently; there are no reports on the role of STIL in bladder cancer. Using public databases; we observed that STIL is highly expressed in bladder cancer and is closely related to the cell cycle. The results of tumor formation experiments on UMUC3 and T24 bladder cancer cell lines indicate that STIL promotes the growth of bladder cancer cells in vivo and in vitro. Mechanistically, the cell cycle after STIL knockout was arrested in the G0/G1 phase; and cell cycle-related proteins (cyclin D1 and CDK2/4/6) were also reduced. RNA-seq results and immunoblotting experiments confirmed that STIL enhanced the PI3K/AKT/mTOR pathway, resulting in increased c-myc expression which ultimately promoted the occurrence and progression of bladder cancer. Our results suggest that STIL may be a promising potential therapeutic target for bladder cancer.

**Abstract:**

SCL/TAL1 interrupting locus (STIL) regulates centriole replication and causes chromosome instability, which is closely related to malignant tumors. The purpose of our study was to investigate the role of STIL in bladder cancer (BC) tumorigenesis for the first time. The public database indicated that STIL is highly expressed and correlated with the cell cycle in BC. Immunohistochemistry staining showed that STIL expression is significantly elevated in BC tissues compared with paracancer tissues. CRISPR-Cas9 gene editing technology was used to induce BC cells to express STIL-specific sgRNA, revealing a significantly delayed growth rate in STIL knockout BC cells. Moreover, cell cycle arrest in the G0/G1 phase was triggered by decreasing STIL, which led to delayed BC cell growth in vitro and in vivo. Mechanically, STIL knockout inhibited the PI3K/AKT/mTOR pathway and down-regulated the expression of c-myc. Furthermore, SC79 (AKT activating agent) partially reversed the inhibitory effects of STIL knockout on the proliferation and migration of BC cells. In conclusion, STIL enhanced the PI3K/AKT/mTOR pathway, resulting in increased expression of c-myc, ultimately promoting BC occurrence and progression. These results indicate that STIL might be a potential target for BC patients.

## 1. Introduction

The incidence of bladder cancer (BC) ranks eleventh among all malignant tumors in the world [1]. BC is divided into two types, the majority being non-muscle-invasive diseases (90%) and the minority being muscle-invasive diseases [2,3]. Non-muscle-invasive BC frequently recurs (47%) [4], which greatly affects patients’ quality of life. Furthermore, muscle-invasive BC has a poor prognosis and a five-year survival rate of <50% [5]. Despite advances in chemotherapy and surgery, the prognosis has not changed significantly, and new entry points for treatment options are urgently needed [6]. Therefore, it is important for BC diagnosis and prognosis to discover novel biomarkers. 

SCL/TAL1 interrupting locus (STIL), a key regulator involved in the regulation of centriole duplication, is an important checkpoint protein in mitosis [7]. Chromosomal instability caused by ectopic centriolar amplification is the main feature of human cancer [8]. Elevated expression of STIL has been reported in various cancers, such as colorectal cancer, pancreatic cancer, gastric cancer, prostate cancer, lung cancer, and nasopharyngeal carcinoma [9,10,11,12,13,14]. STIL promotes the development of many cancers; however, there is no detailed investigation of STIL in BC.

In our study, we found that STIL was abnormally expressed in BC patients and predicted a poor prognosis. Moreover, STIL knockout markedly blocked proliferation and migration of BC cells in vitro and inhibited proliferation of tumor in vivo, and triggered cell cycle arrest in the G0/G1 phase. Mechanistically, STIL promoted the PI3K/AKT/mTOR pathway and increased c-myc expression, thereby facilitating BC progression.

## 2. Materials and Methods

### 2.1. Tissue Microarray and Immunohistochemistry

TMA (tissue microarrays) (product code: HBlaU079Su01, Outdo Biotech, Shanghai, China) included 63 BC tissues and 16 paracancer tissues. The samples were collected from May 2007 to January 2011. TMA slices were dehydrated with different concentrations of gradient alcohol. After being made transparent with a transparent agent (xylene), the slices were soaked in 3 pots of paraffin at 60 °C one by one for 1 h and finally embedded and sectioned. Antigen repair was performed in an electrochromic oven with a repair solution (0.01 mM EDTA buffer, pH 9.0). Three-percent hydrogen peroxide was added to block endogenous peroxidase, and goat serum was blocked at room temperature for 30 min. Then, the slices were incubated overnight (12 h) with a primary antibody. Next, the slices were incubated with the second antibody at 37 °C for 30 min, and then with a freshly prepared DAB chromogenic solution. After redyeing with hematoxylin, the slices were dehydrated, sealed, scanned, and photographed under a microscope. Immunohistochemical scores were performed independently by two experienced pathologists. In general, the expression of STIL was evaluated on the criteria of the intensity and positive staining rate of immunostaining of the tumor tissue. The positive signal was brownish yellow or brown. The staining intensity was scored as 0, 1, 2, and 3. The positive staining rate score was defined as 0 (0%), 1 (1–25%), 2 (26–50%), 3 (50–75%), and 4 (75–100%). Histoscore was calculated by multiplying the staining intensity score and the staining positive rate score.

### 2.2. Cell Culture

The human BC cell lines include the UMUC3, EJ, J82, T24, and SCABAR cell lines, HEK293T cell line, and normal human bladder cell line SV-HUC-1. These cells were donated by the Stem Cell Bank, CASS, China. One hundred U/mL penicillin, 10% fetal bovine serum (Invitrogen, Shanghai, China), and 0.1 g/mL streptomycin sulfate were added to RPMI medium 1640 (Invitrogen, Shanghai, China) or DMEM high glucose medium (Gibco, Shanghai, China). SV-HUC-1, EJ, and T24 cells were cultured in mixed RPMI medium 1640; UMUC3, J82, SCABAR, and HEK293T cells were cultured in mixed DMEM high-glucose medium. All cells were incubated at 37 °C with 5% CO_2_ and 95% air.

### 2.3. STIL Knockout in UMUC3 and T24 Cells

We performed STIL knockout in UMUC3 and T24 cells with the CRISPR-Cas9 system. In this system, small guide RNAs (sgRNAs) of STIL were cloned into lenti-v2 (Addgene, 92062, Shanghai, China). HEK293T cells were co-transfected with recombinant lenti-CRISPR-v2 and package plasmid to generate lentivirus for 48 h. The supernatant was collected and added to UMUC3 and T24 cells, and incubated for 36 h; subsequently, 1000 ng/mL puromycin was added for 4 days. We used a limited dilution method to obtain the cloned cells (UMUC3 and T24 cells were immobilized on five 96-well plates and grown for 3 weeks). We screened monoclonal cells by Western blotting to obtain accurate STIL knockout cells. The sgRNA sequences were sgRNA-F, 5′-caccGGGGTTATTTCTAGGCATTC-3′; sgRNA-R: 5′-aaacGAATGCCTAGAAATAACCCC-3′.

### 2.4. CCK-8 Assay

The cells with STIL knockout were inoculated into 96-well plates with 2 × 10^3^ cells per well. The absorbance of 96-well plates was measured on days 1, 2, 3, 4, 5, and 6, respectively. Ten μL of CCK-8 solution (BS350A, biosharp, Hefei, China) was added to each well and cultured in a cell incubator for 3 h. The optical density was measured at 450 nm using a multifunctional enzyme marker (PE Enspire, PerkinElmer, Singapore).

### 2.5. Cell Colony Formation

The cells were inoculated into 6-well plates at a density of 1.2 × 10^3^ cells per well and cultured for about 3 weeks until clearly visible to the naked eye. Finally, they were dyed with 0.3% crystal violet (dissolved by anhydrous ethanol), washed gently with water, and photographed.

### 2.6. Transwell Migration Assays

Medium containing 20% fetal bovine serum was added to the lower chamber of the 24-well plates, and 5 × 10^4^ cells with serum-free medium were added to the upper chamber of a transwell chamber (Thermo Fisher Scientific, Shanghai, China) to culture for 24 h. The migrated cells were fixed with 4% paraformaldehyde (biosharp, Shanghai, China), stained with 0.3% crystal violet, photographed, and counted with an inverted microscope.

### 2.7. Soft Agar Assay

One-point-four-percent low-melting-point agarose (Promega, Madison, WI, USA) was added to 6-well plates and solidified at room temperature. Ten thousand cells were mixed with 0.7% low-melting-point agarose and overlaid on 1.4% low-melting-point agarose. After solidifying at room temperature again, they were put into an incubator and incubated for about 4 weeks before being photographed under a microscope.

### 2.8. Cell Cycle Assay

We used a cell cycle staining kit (Multisciences, 70-CCS012, Shanghai, China). After collecting 5 × 10^5^ cells, the cells were washed with PBS. Then, 1 mL of DNA staining solution and 10 μL of osmotic solution were added to the cells. After incubating at room temperature for 30 min, the cells were detected on a flow cytometer (CytoFlex S, Beckman Coulter, Wuhan, China) with the lowest flow rate.

### 2.9. The EDU (5-Ethynyl-2-deoxyuridine) Assay

EDU cell proliferation detection kit (C0071S, Shanghai, China) was purchased from Beyotime. Cells were incubated with EDU working solution for 2 h, then fixed with 4% paraformaldehyde and treated with permeabilization solution (P0097, Immunostaining Strong Permeabilization Solution, Beyotime, Shanghai, China) for 30 min at room temperature. According to the protocol of the manufacturer, cells were incubated in click reaction solution (CuSO_4_, Click Reaction Buffer, Click Additive Solution, Azide 488) at room temperature in the dark for 30 min. Nuclei staining was conducted with Hoechst (33342, Beyotime, Shanghai, China) reagent. After staining, the cells were observed under a confocal fluorescence microscope or prepared as cell suspension samples for flow cytometry.

### 2.10. Immunofluorescence Assay

The Ki67 cell proliferation kit (E607238-0100, sangon, Shanghai, China) was used to measure cell proliferation. For specific steps, please refer to the instruction manual. Briefly, cells were incubated with the diluted primary antibody Ki67 overnight at 4 °C and incubated with the fluorescent-labeled secondary antibody at 37 °C for 30 min. Nuclei were then counterstained with 4′,6-diamidino-2-phenylindole (DAPI). Finally, immunofluorescence staining was observed under an inverted fluorescence microscope.

### 2.11. RT-qPCR

RNA was extracted from cells using trizol reagent (Invitrogen, Shanghai, China); then, reverse transcription was performed using the cDNA reverse transcription kit (Toyobo Life Science, Shanghai, China) to synthesize cDNA. RT-qPCR was performed using the SYBR Green PCR kit (TSE201, Tsingke Biotechnology, Wuhan, China) and a Bio-Rad CFX96 PCR system to detect CT values. Primer sequences are in Supplementary Table S1.

### 2.12. Western Blotting

Protease inhibitors were added to the RIPA buffer (Beyotime, Wuhan, China) to lyse cells on ice and eventually extract proteins. The proteins were electrophoretic in 8–15% SDS-PAGE gel and transferred to PVDF membrane (IPVH00010, Sigma-Aldrich, Shanghai, China), after which they were sealed with blocking buffer (P30500, Ncm Biotech, Suzhou China) for 15 min. The membrane was incubated overnight in the designated primary antibody at 4 °C. After washing with TBST, the membrane was incubated with the corresponding secondary antibody at room temperature for 1 h. Subsequently, the membrane was imaged on a chemiluminescence image analysis system (Tanon 5200, Wuhan, China). STIL antibodies were purchased from Santa (SC-271910, Dallas, TX, USA). Original blots see File S1.

### 2.13. Tumor Xenografts

The experimental plan was approved by the Animal Biosafety Level III Laboratory of Wuhan University. We injected cells via syringe into the backs of nude mice living in a pathogen-free environment (6 × 10^6^ cells/nude). The nude mice were purchased from China Shanghai Laboratory Animal Research Center. Tumor volume was measured every 3 days after surgery and calculated by the formula: length × width × height × π/6. On the 26th postoperative day, the tumors of mice were collected, photographed, and weighed.

### 2.14. Statistical Analysis

SPSS 25.0 was used for statistical analysis. All experiments were performed at least 3 times. One-way ANOVA was used for three or more groups. A two-sided Student’s t-test was used for the significance of the difference between the two groups. *p* < 0.05 was considered statistically significant (* *p* < 0.05, ** *p* < 0.001, and *** *p* < 0.001).

### 2.15. RNA-Seq Analysis of N-butyl-N-(4-hydroxybutyl) Nitrosamine (BBN)-Treated Bladder Cancer Mouse Models

We obtained the raw sequencing data from the NCBI SRA Database with Bioproject: PRJNA587619 [15]. STAR was used to align RNA sequencing reads [16], and featureCounts was used for quantification of gene and transcript levels [17]. And differentially expressed genes were screened between the BBN-treated group and control group by R package Deseq2 [18].

## 3. Results

### 3.1. STIL Was Highly Expressed in BC and Was Inextricably Intertwined with the Cell Cycle

**Figure 1:**
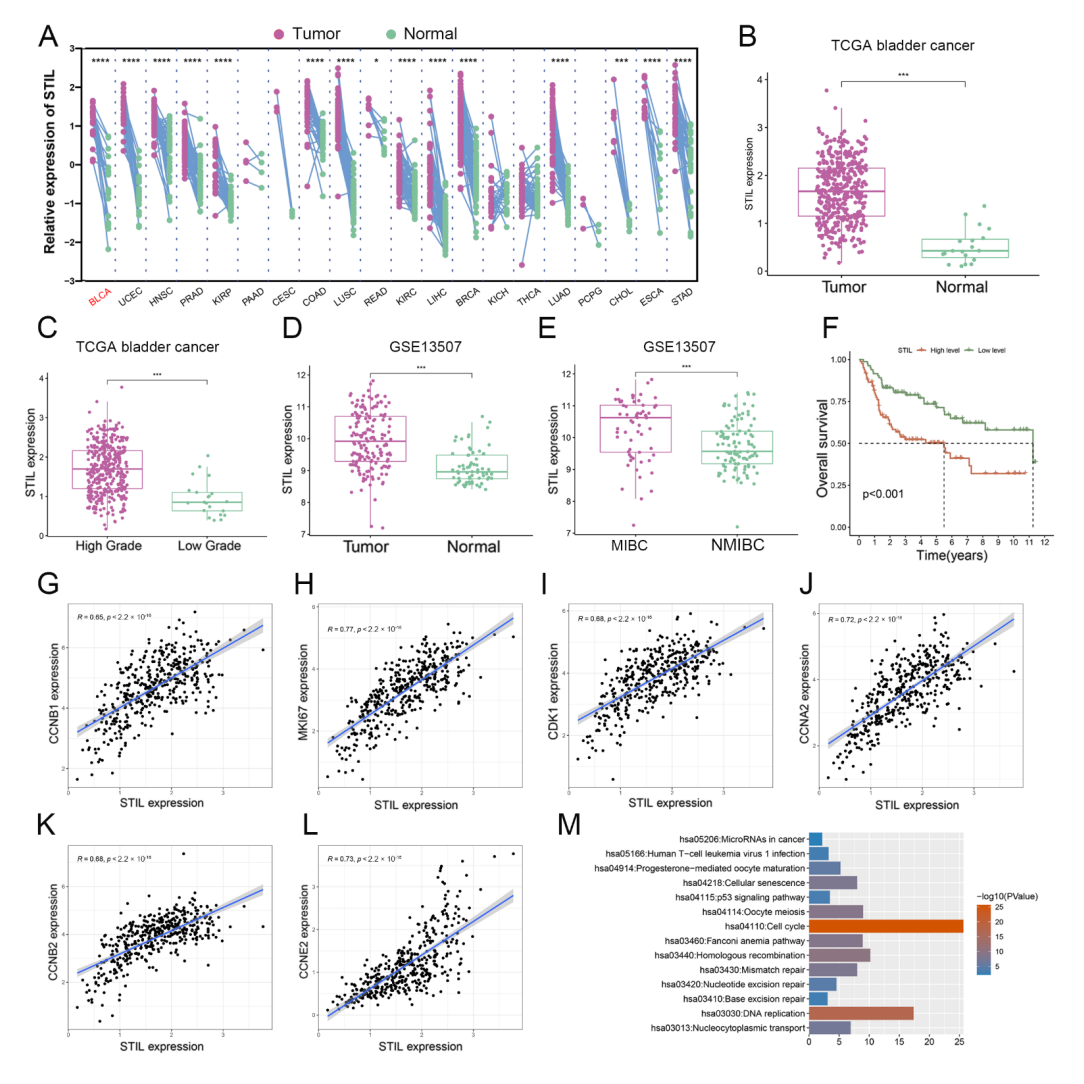
STIL was highly expressed in BC and was inextricably intertwined with the cell cycle. (**A**) Paired samples grouped by cancer from the TCGA show STIL expression in human cancers. Each point represents one sample. *p*-values are based on a two-tailed Student’s *t*-test. (**B**) Gene expression of STIL in BC and normal tissues based on TCGA database. (**C**) Gene expression of STIL in high-grade and low-grade BC tissues based on TCGA database. (**D**) Gene expression of STIL in BC and normal tissues based on the microarray data GSE13507. (**E**) Gene expression of STIL in MIBC and NMIBC based on the microarray data GSE13507. (**F**) The Kaplan–Meier plot shows the overall survival for STIL expression based on the GSE13507. (**G**–**L**) A correlation of STIL expression with proliferation marker MKI67 and the genes regulating the cell cycle, including CCNB1, CDK1, CCNA2, CCNB2, and CCNE2. (**M**) KEGG pathway enrichment results for the genes significantly correlated with STIL. STIL, SCL/TAL1 interrupting locus; BC, bladder cancer; MIBC, muscle-invasive bladder cancer; NMIBC, non-muscle invasive bladder cancer ( * *p* < 0.05, *** *p* < 0.001 and **** *p* < 0.0001).

To analyze the expression levels of STIL in various tumors, we analyzed TCGA RNA sequencing (RNA-seq) data and found that STIL expression was significantly elevated in various cancer types, including bladder urothelial carcinoma (BLCA), uterine corpus endometrial carcinoma (UCEC), head and neck squamous cell carcinoma (HNSC), prostate adenocarcinoma (PRAD), kidney renal papillary cell carcinoma (KIRP), colon adenocarcinoma (COAD), lung squamous carcinoma (LUSC), rectum adenocarcinoma (READ), clear cell renal cell carcinoma (KIRC), liver hepatocellular carcinoma (LIHC), breast invasive carcinoma (BRCA), lung adenocarcinoma (LUAD), cholangiocarcinoma (CHOL), esophageal carcinoma (ESCA), and stomach adenocarcinoma (STAD) (Figure 1A). To further verify the expression levels of STIL in bladder cancer (BC), we analyzed the RNA-seq datasets of several BC tissues from TCGA. The results showed that STIL mRNA levels were significantly higher in BC tissues than in normal bladder tissues, and significantly higher in high-grade BC than in low-grade BC (Figure 1B,C). Similarly, in the GSE13507 database, STIL mRNA leveler was higher in BC tissues than normal bladder tissues, and higher in muscle-invasive bladder cancer (MIBC) than non-muscle invasive bladder cancer (NMIBC) (Figure 1D,E). In addition, BC patients with high expression of STIL had poor prognoses (Figure 1F). Interestingly, the results showed that cell cycle-related genes (CCNB1, MKI67, CDK1, CCNA2, CCNB2, CCNE2) were significantly positively correlated with STIL (Figure 1G–L). The results from the KEGG pathway enrichment analysis showed that the genes correlated with STIL were significantly enriched in the cell cycle (Figure 1M). Marina Degoricija et al. checked the expression of the STIL gene in the most common mouse model of bladder cancer, the so-called BBN model in already published papers in which RNA-seq analysis was carried out on that model [15]. On these RNA-seq data, we found that STIL mRNA expression was significantly up-regulated in BBN-induced bladder cancer tissues, compared with control samples. Moreover, some other genes including AKT, c-myc, MCM4, and EIF2S2 were also significantly elevated in cancer tissues. These results were mostly consistent with our findings from human bladder cancer. We presented our data in the Supplementary Materials, Figure S4.

### 3.2. Expression of STIL Was Significantly Up-Regulated in BC

**Figure 2:**
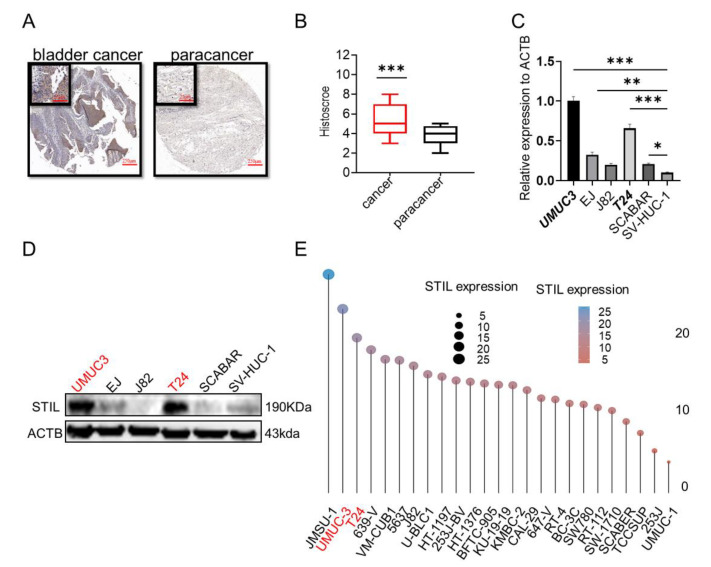
The expression of STIL in BC was higher than in the normal bladder. (**A**,**B**) Tissue microarray analysis of STIL expression in 16 paracancer tissues and 63 BC tissues. (**A**) Representative images of BC and paracancer tissues. (**B**) Histoscore of BC and paracancer tissues. (**C**) The mRNA levels of STIL in five BC cell lines (UMUC3, EJ, J82, T24, SCABAR) and the normal bladder cell line (SV-HUC-1). (**D**) Protein levels of STIL in the UMUC3, EJ, J82, T24, SCABAR, and SV-HUC-1 cells. (**E**) Expression distribution of STIL in different cell lines. The horizontal coordinate, dot size, and different colors represented levels of expression. The BC cell lines mRNA expression matrix was collected from CCLE (https://portals.broadinstitute.org/ccle, Accessed on 2 August 2022). This result was obtained by R (v4.0.3) package ggplot2 (v3.3.3) (ACTB, β-actin) (* *p* < 0.05, ** *p* < 0.01, and *** *p* < 0.001).

Immunohistochemical staining was performed on 63 BC tissues and 16 paracancer tissues from the tissue microarray; the findings revealed that the expression of STIL was significantly up-regulated in BC tissues compared to paracancer tissues (Figure 2A,B). Subsequently, we examined mRNA and protein levels in the normal bladder cell line (SV-HUC-1) and five BC cell lines; results showed that the expression levels of STIL in BC cells were significantly higher than in normal cells (Figure 2C,D). The UMUC3 and T24 cell lines showed the highest STIL expression (Figure 2E). Therefore, UMUC3 and T24 cells were used in the subsequent cell experiments in our study.

### 3.3. Knockout of STIL Inhibited BC Tumorigenesis In Vitro

**Figure 3:**
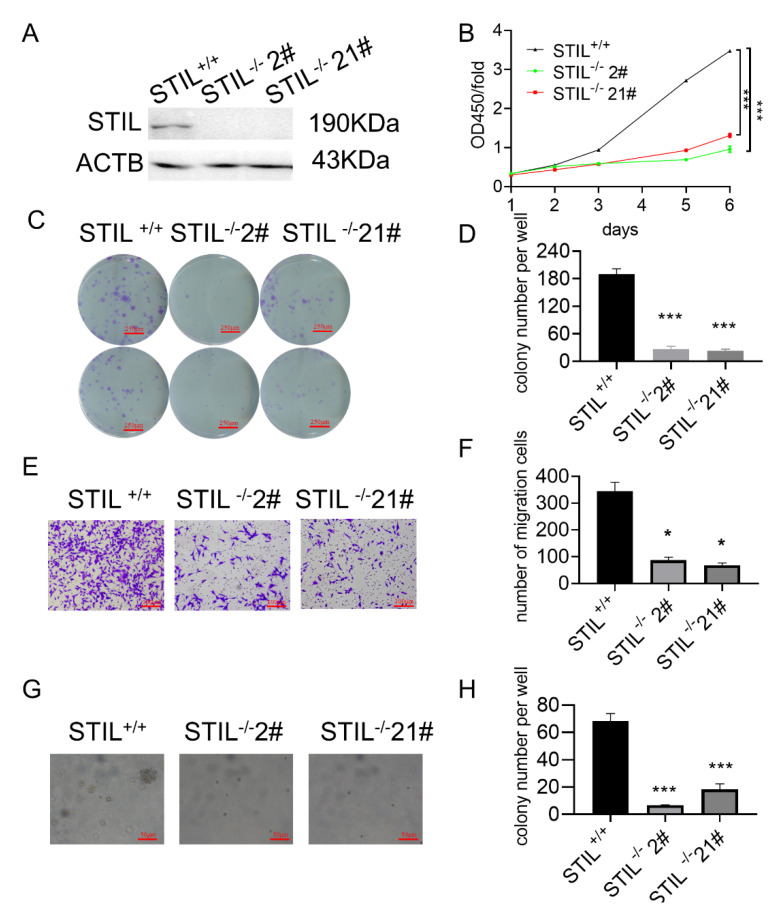
In the UMUC3 cell line, knockout of STIL inhibited BC tumorigenesis in vitro. (**A**) After screening by Western blot, the exact STIL knockout cells (STIL^−/−^ 2# and STIL^−/−^ 21#) were obtained. (**B**) CCK-8 assay. (**C**) Representative microscope images and (**D**) quantitative analysis of colony formation assays. (**E**) Representative microscope images and (**F**) quantitative analysis of transwell migration assay. (**G**) Representative microscope images and (**H**) quantitative analysis of soft agar assay. All experiments were conducted with the following groups: control group (STIL^+/+^ cells) and experimental groups (STIL^−/−^ 2# cells and STIL^−/−^ 21# cells). CCK-8 assay, Cell Counting Kit-8 assay (* *p* < 0.05 and *** *p* < 0.001).

We performed a CRISPR-Cas9-based STIL knockout assay in the UMUC3 cell line. The STIL knockout cells, especially STIL^−/−^ 2# and STIL^−/−^ 21#, were screened for the best knockout efficiency by Western blot (STIL^+/+^: wild-type cell. STIL^−/−^: STIL knockout cell) (Figure 3A). In the Cell Counting Kit-8 (CCK-8) assay, STIL knockout revealed remarkable inhibition of cell proliferation (Figure 3B). The colony formation assay showed that STIL knockout significantly reduced cell proliferation (Figure 3C,D). Similarly, cell migration was significantly reduced by STIL knockout in the transwell migration assay (Figure 3E,F). Moreover, the colony size and the number on soft agar, which always indicate cell growth, were reduced in STIL knockout cells (Figure 3G,H). We also performed knockout of STIL in the T24 cell line using CRISPR-Cas9 technology, and finally obtained STIL^−/−^ 8# and STIL^−/−^16# (Figure S1A). Similarly, we performed a CCK-8 assay (Figure S1B), colony formation assay (Figure S1C,D), transwell migration assay (Figure S1E,F), and soft agar assay (Figure S1G,H). The results show that the ability of T24 cell to proliferate and migrate was decreased after STIL knockout.

### 3.4. Cell Cycle of STIL Knockout Cells Was Arrested in the G0/G1 Phase

**Figure 4:**
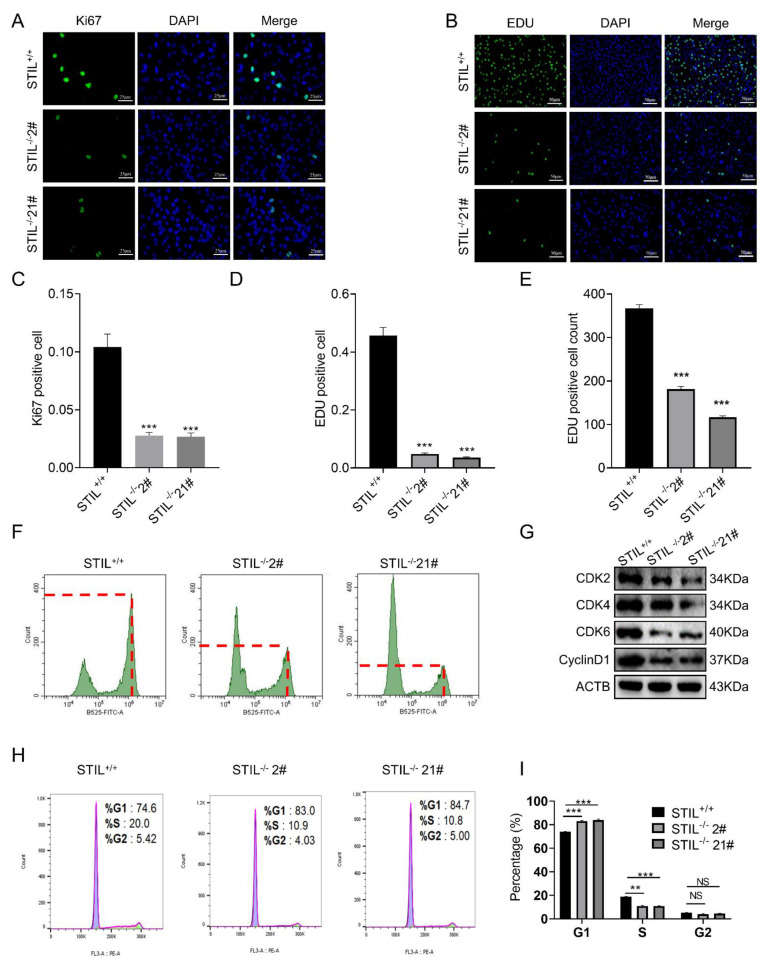
In the UMUC3 cell line, STIL played a vital role in the proliferation and cell cycle of BC. Representative microscope images of (**A**) Ki67 staining and (**B**) EDU staining. The green color represents Ki67 and EDU and the blue color represents DAPI. The quantitative analysis of (**C**) Ki67 staining and (**D**) EDU staining. (**F**) Representative images of flow cytometry for EDU staining and (**E**) quantitative analysis of EDU positive cell count. Ten thousand cells were counted in each sample. The ordinate of the second peak is the number of EDU-positive cells, and the fluorescence intensity (abscissa of the graph) was 10^6^ at this point. (**G**) Representative images of Western blotting of cyclin D1 and cell cycle-related proteins CDK2/4/6. (**H**) Representative images of flow cytometry for propidium iodide (PI) staining and (**I**) quantitative analysis of cell cycle phase. EDU, 5-ethynyl-2-deoxyuridine (** *p* < 0.01 and *** *p* < 0.001; NS, no significance).

To understand the mechanisms of how STIL knockout inhibits BC tumorigenesis, we examined the cell cycle progression of STIL knockout UMUC3 cells and T24 cells. The results showed that STIL knockout markedly reduced the percentage of Ki67 positive cells (Figure 4A,C and Figure S2A,C) and 5-ethynyl-2-deoxyuridine (EDU) positive cells (Figure 4B,D and Figure S2B,D). Flow cytometry analysis showed that proliferation cells (EDU positive cells) were significantly reduced after STIL knockout (Figure 4E,F). To explore the relationship of STIL to the cell cycle in proliferation, we assessed changes in the cyclin-dependent kinases (CDKs). At the protein level, CDK2/4/6 and cyclin D1 were decreased after STIL knockout (Figure 4G and Figure S2E). Flow cytometry analysis showed that STIL knockout cells were stagnant in the G0/G1 phase to prevent cell proliferation (Figure 4H,I and Figure S2F,G).

### 3.5. The Proliferative Function of STIL Was Confirmed by the Xenotransplantation Model

**Figure 5:**
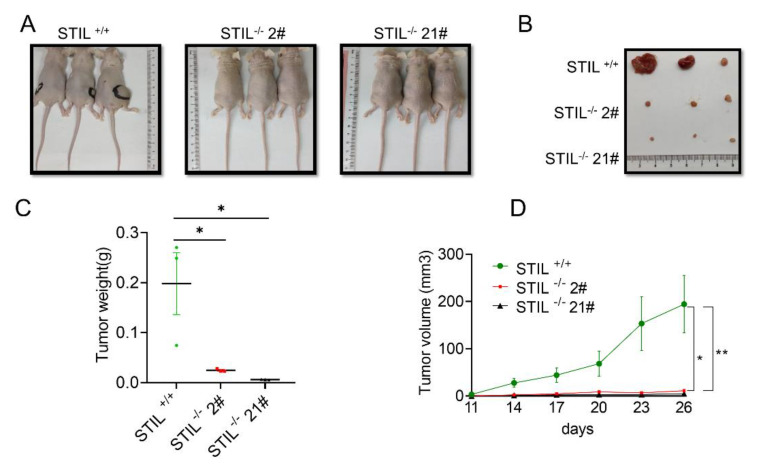
Knockout of STIL played a role in inhibiting tumor growth in vivo. The nude mice were terminated 26 days after cell injection. (**A**) Images of nude mice. (**B**) Images of solid tumors of nude mice. (**C**) Point diagram of the weight of each group of tumors. (**D**) Tumor volume growth curve of nude mice. The nude mice were observed every 3 days after the injection of the cells, and tumor entities were detected from day 11 to day 26 (* *p* < 0.05 and ** *p* < 0.01).

To demonstrate the function of STIL in vivo, we constructed a xenograft model. STIL^+/+^, STIL^−/−^, 2#, and STIL^−/−^ 21# cells were implanted into nude mice. On the 26th day, tumors were removed from the nude mice. The solid tumor volume of STIL^+/+^ was significantly larger than that of STIL^−/−^ 2# and STIL^−/−^ 21# (Figure 5A–C). In addition, through dynamic observation of tumor growth, it was found that the tumor growth of the STIL knockout cells was slowed (Figure 5D). These results suggested that STIL knockout has a dramatic effect on the regulation of BC tumorigenesis in vivo.

### 3.6. STIL Knockout Down-Regulated the PI3K/AKT/mTOR/c-myc Signaling Pathway

**Figure 6:**
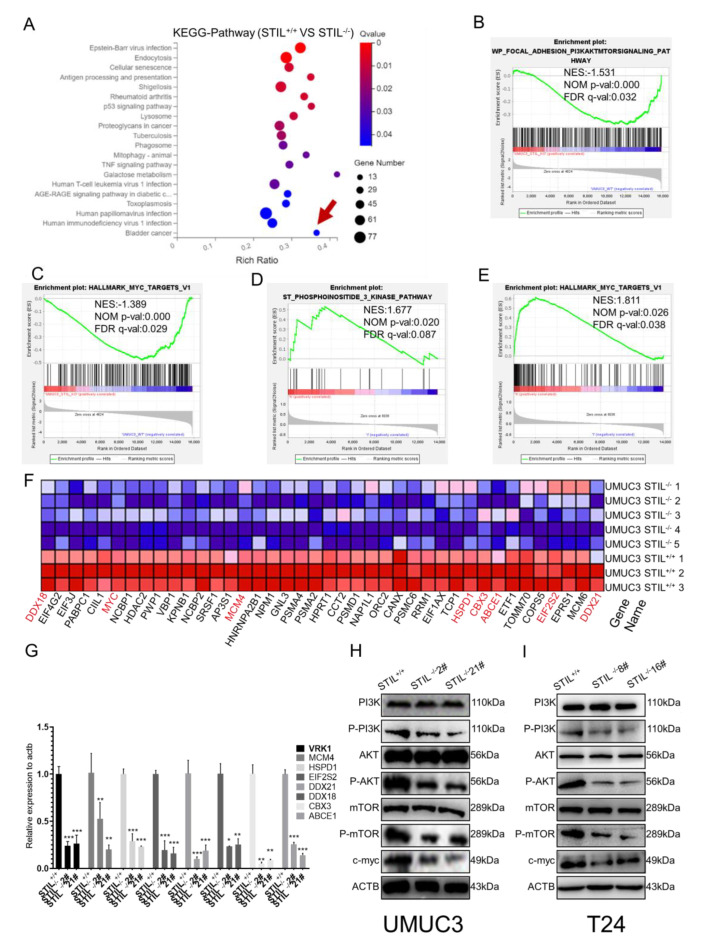
STIL knockout down-regulated the PI3K/AKT/mTOR/c-myc signaling pathway. (**A**) The RNA-seq analysis of STIL knockout and wild-type cells: we used KEGG pathway enrichment analysis in STIL^+/+^ and STIL^−/−^ BC cells. GSEA identified (**B**) PI3K/AKT/mTOR pathway-related gene sand (**C**) c-myc sets enriched in the STIL^−/−^ BC cells. Through the website www.broadinstitute.org/gsea, we found that the (**D**) PI3K pathway and (**E**) c-myc were enriched in BC patients. (**F**) The difference in expression of c-myc downstream molecules is represented by a heat map based on our RNA sequencing analysis of STIL^+/+^ and STIL^−/−^ cells. Red/green colors represent higher/lower expression. (**G**) RT-qPCR analysis of downstream genes of c-myc (VRK1, MCM4, HSPD1, EIF2S2, DDX21, DDX18, CBX3, ABCE1). After STIL knockout, these downstream molecules were down-regulated. (**H**,**I**) Western blotting detected levels of PI3K/p-PI3K, AKT/ p-AKT, mTOR/ p-mTOR, and c-myc in UMUC3 and T24 cells. GSEA, gene set enrichment analysis (* *p* < 0.05, ** *p* < 0.01, and *** *p* < 0.001).

To further disclose the potential mechanism behind STIL-driven malignant behaviors in bladder cancer, we analyzed the differentially expressed genes in STIL-deficient UMUC3 cells and wild-type UMUC3 cells. The results of RNA-seq showed that the PI3K/AKT/mTOR/c-myc signaling pathways were significantly decreased in STIL-deficient cells compared to the wild-type, as found through GSEA analysis based on KEGG pathways (Figure 6A–C). We also checked the RNA sequencing of 412 BC patients from the TCGA database and divided the patients into a high-expression group and a low-expression group according to median STIL expression. We found that PI3K/AKT/mTOR/c-myc signaling pathways were significantly enriched in the high-expression group (Figure 6D,E). Our RNA-seq analysis results showed that the downstream molecules of the transcription factor c-myc (including MCM4, HSPD1, EIF2S2, DDX21, DDX18, CBX3, ABCE1, etc.) were significantly down-regulated in BC cells after STIL knockout (Figure 6F). To confirm this result, we verified by RT-qPCR, showing that the expression of these molecules was significantly decreased after STIL knockout (Figure 6G). To further demonstrate the relationship between STIL, the PI3K/AKT/mTOR pathway, and c-myc in BC, we performed an immunoblotting analysis. PI3K/AKT/mTOR protein levels did not fluctuate significantly after STIL knockout, but p-PI3K/p-Akt/p-MTOR/c-myc protein levels significantly decreased in STIL knockout BC cells (Figure 6H,I).

### 3.7. SC79 Treatment Partially Reversed the Effects of STIL Knockout on Cell Tumorigenesis

**Figure 7:**
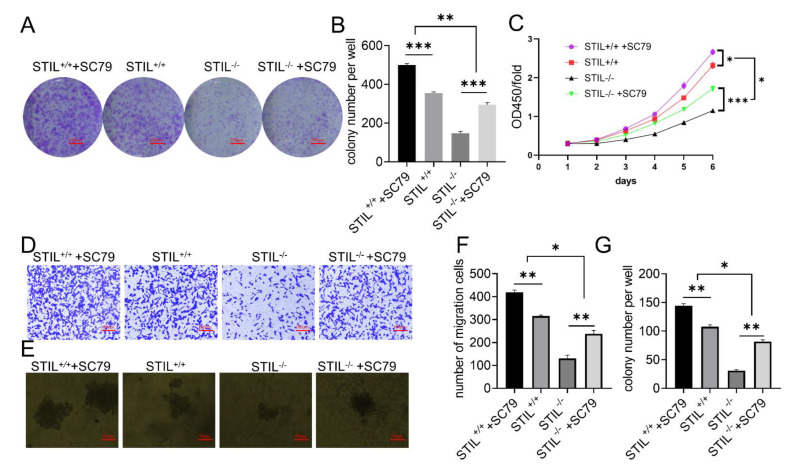
In the UMUC3 cell line, SC79 treatment partially reversed the effect of STIL knockout on cell tumorigenesis. (**A**) Representative microscope images and (**B**) quantitative analysis of colony formation assays. (**C**) Cell proliferation curves of CCK-8 assays. (**D**) Representative microscope images and (**F**) quantitative analysis of transwell migration assay. (**E**) Representative microscope images and (**G**) quantitative analysis of soft agar assay. In all the above experiments, four groups of cells were tested simultaneously (STIL^+/+^+SC79, STIL^+/+^, STIL^−/−^, STIL^−/−^+SC79) (* *p* < 0.05, ** *p* < 0.01, and *** *p* < 0.001).

To further verify the relation between PI3K/AKT/mTOR/c-myc signaling pathway and STIL-driven malignant behaviors in BC, we activated the AKT pathway in STIL^+/+^ and STIL^−/−^ cell lines with SC79 (AKT activating agent). After activation of the PI3K/AKT/mTOR pathway by SC79, the STIL-driven inhibition of migration and proliferation was partially reversed, as shown by colony formation assays (Figure 7A–C and Figure S3A–C). Next, we performed a transwell migration assay; the results of this assay were similar to the colony formation experiment (Figure 7D,F and Figure S3D,F). In addition, the soft agar assay also showed that SC79 increased cell proliferation in STIL^−/−^ cells more than in STIL^+/+^ cells (Figure 7E,G and Figure S3E,G). Effectively, after activation of the PI3K/AKT/mTOR pathway, the elevated tumorigenesis levels in STIL knockout BC cells were partially restored.

## 4. Discussion

Previous studies have shown that STIL causes abnormal centriole expansion, which, in turn, leads to chromosomal instability [7]. Chromosomal instability is the hallmark of many cancers [8]. STIL is considered to be an oncogene whose expression is elevated in many types of cancers [9,10,11,12,13]. However, there is little in the literature on the question of STIL in BC. Our study found that the mRNA expression levels of STIL were significantly elevated and were positively correlated with the cycle-related gene (CCNB1, CDK1, CCNA2, CCNB2, and CCNE2) in BC. In addition, BC patients with high expression of STIL had poor prognoses. Demonstrated by microarray detection and immunohistochemistry, we confirmed an increase in STIL expression in clinical specimens. Previous studies have suggested that STIL is critical for cancer cell migration [19]. Our experimental results show that STIL has similar results in BC: STIL knockout inhibited BC cells proliferation and migration in vitro and blocked proliferation of tumor in vivo. These results strongly showed that STIL is related to the development and occurrence of BC. STIL is a cell-proliferation-related gene involved in cell cycle regulation [20]. We analyzed cell proliferation by detecting cell proliferation markers (EDU, Ki67), as well as observing changes in cell cycle and cycle-related proteins. 

According to previous studies, the ability of the G0/G1 phase to enter the S phase is mainly determined by CDKs, including CDK2/4/6 [21,22]. Cyclin D and CDK4/6 are highly related and readily form conjugates. By activating CDK2, the cyclin D-CDK4/6 complex promotes DNA replication and cell proliferation [22]. In many tumors, reductions in CDK2/4/6 and cyclin D1 often coexist with G0/G1 cell cycle arrest [23,24,25]. And some studies have shown that STIL is closely related to G1 phase arrest [26]. A similar phenomenon emerged in our study: STIL knockout resulted in cell cycle arrest in the G0/G1 phase in BC cells. At the protein level, CDK2/4/6 and cyclin D1 were decreased in STIL knockout BC cells. STIL knockout showed different blockade phases in different tumor types, and reduced the proliferation of cervical and colon cancer cells by inhibiting Cyclin B1/CDK1, which, in turn, induced cell cycle arrest in the G2/M phase [11,27,28]. 

To explore the effects of STIL on proliferation in vivo, we used a xenograft model and confirmed that STIL knockout inhibited the growth of xenograft tumor cells. Our RNA sequencing results and gene set enrichment analysis (GSEA) showed a significant decrease in PI3K/AKT/MTOR pathway enrichment and c-myc target enrichment after STIL knockout. Heat map analysis showed that the downstream molecules of c-myc were significantly down-regulated in STIL knockout BC cells. RT-qPCR and Western blotting were used to confirm that the downstream p-PI3K/p-AKT/p-mTOR/c-myc pathway was down-regulated after STIL knockout. These results suggest that STIL plays a cycle-related gene vital role in BC through the PI3K/AKT/mTOR pathway and c-myc. Significant research has shown that the PI3K/AKT signaling pathway plays a significant role in promoting invasion, migration, proliferation, and other malignant characteristics of human cancers [29,30]. C-myc, as a proto-oncogene, is fundamental to regulating cell proliferation [31,32]. Several studies have documented that the activation of c-myc is mediated by the AKT/mTOR pathway [33,34,35]. Furthermore, c-myc is up-regulated in many tumors and is important for proliferation [36,37]. Additionally, c-myc is crucial to the cell cycle progression of tumor cells [38]. C-myc acts as a transcription factor to stimulate cell cycle progression and cell proliferation [39,40]. It has been proposed that c-myc regulates cell cycle progression through CDK2/4 and cyclin D1 [41,42]. These previous studies are consistent with our findings. Thus, we conclude that STIL reduces c-myc through the PI3K/AKT/ mTOR signaling pathway, leading to a decrease in CDK2/4/6 and cyclin D1, thereby causing cell cycle arrest in the G0/G1 phase and ultimately inhibiting cell proliferation. Therefore, in the future, it may be possible to inhibit tumor proliferation by inhibiting STIL, reducing c-myc, and arresting the G0/G1 phase. Additionally, PI3K/AKT/mTOR signal transduction is modulated by SC79 in BC cells, which significantly reduces the down-regulation of STIL knockout. We proved that changes in STIL drive fluctuations in c-myc, which affect the cell cycle. However, we still do not know the direct target of STIL; we will address this question in future research.

In summary, we are the first to confirm that STIL may promote development in BC. Our results suggest that STIL is strongly associated with prognosis in BC patients and mediates c-myc through the PI3K/AKT/ mTOR signaling pathway, ultimately promoting the BC cell cycle, proliferation, invasion, and metastasis. These findings may shed new light on the role of STIL in human malignancies and provide new targets for treating BC.

## 5. Conclusions

STIL enhanced the PI3K/AKT/mTOR pathway, resulting in increased expression of c-myc, ultimately promoting BC occurrence and progression. These results indicate that STIL may be a potential target for treating BC.

## Data Availability

The data presented in this study are openly available in GEO, reference number GSE211756. The data can be found here: https://www.ncbi.nlm.nih.gov/geo/query/acc.cgi?acc=GSE211756.

## Supplementary Materials

The following supporting information can be downloaded at: https://www.mdpi.com/article/10.3390/cancers14235777/s1, Table S1: real-time quantitative PCR primer sequences. Figure S1. In the T24 cell line, knockout of STIL inhibited BC tumorigenesis in vitro. Figure S2. In the T24 cell line, STIL played a vital role in the proliferation and cell cycle of BC. Figure S3. In the T24 cell line, SC79 treatment partially reversed the effect of STIL knockout on cell tumorigenesis. Figure S4. STIL and some other genes mRNA expression was significantly upregulated in BBN induced bladder cancer tissues, compared with control samples. The original WB can be found in File S1.

## References

[1] China National Highways (2019). Chinese guidelines for diagnosis and treatment of urothelial carcinoma of bladder 2018 (English version). Chin. J. Cancer Res..

[2] Colombel M., Soloway M., Akaza H., Böhle A., Palou J., Buckley R., Lamm D., Brausi M., Witjes J.A., Persad R. (2008). Epidemiology, Staging, Grading, and Risk Stratification of Bladder Cancer. Eur. Urol. Suppl..

[3] Prout G.R., Barton B.A., Griffin P.P., Friedell G.H. (1992). Treated history of noninvasive grade 1 transitional cell carcinoma. The National Bladder Cancer Group. J. Urol..

[4] Messing E.M., Tangen C.M., Lerner S.P., Sahasrabudhe D.M., Koppie T.M., Wood D.P., Mack P.C., Svatek R.S., Evans C.P., Hafez K.S. (2018). Effect of Intravesical Instillation of Gemcitabine vs Saline Immediately Following Resection of Suspected Low-Grade Non-Muscle-Invasive Bladder Cancer on Tumor Recurrence: SWOG S0337 Randomized Clinical Trial. JAMA.

[5] Knowles M.A., Hurst C.D. (2015). Molecular biology of bladder cancer: New insights into pathogenesis and clinical diversity. Nat. Rev. Cancer.

[6] Soloway M.S. (2013). Bladder cancer: Lack of progress in bladder cancer--what are the obstacles?. Nat. Rev. Urol..

[7] Arquint C., Nigg E.A. (2016). The PLK4-STIL-SAS-6 module at the core of centriole duplication. Biochem. Soc. Trans..

[8] Arquint C., Sonnen K.F., Stierhof Y.D., Nigg E.A. (2012). Cell-cycle-regulated expression of STIL controls centriole number in human cells. J. Cell Sci..

[9] Pradhan T., Kumar V., Surya H.E., Krishna R., John S., Jissa V.T., Anjana S., Chandramohan K., Nair S.A. (2021). STIL Endows Oncogenic and Stem-Like Attributes to Colorectal Cancer Plausibly by Shh and Wnt Signaling. Front. Oncol..

[10] Kasai K., Inaguma S., Yoneyama A., Yoshikawa K., Ikeda H. (2008). SCL/TAL1 interrupting locus derepresses GLI1 from the negative control of suppressor-of-fused in pancreatic cancer cell. Cancer Res..

[11] Wang J., Zhang Y., Dou Z., Jiang H., Wang Y., Gao X., Xin X. (2019). Knockdown of STIL suppresses the progression of gastric cancer by down-regulating the IGF-1/PI3K/AKT pathway. J. Cell. Mol. Med..

[12] Wu X., Xiao Y., Yan W., Ji Z., Zheng G. (2019). The human oncogene SCL/TAL1 interrupting locus (STIL) promotes tumor growth through MAPK/ERK, PI3K/Akt and AMPK pathways in prostate cancer. Gene.

[13] Erez A., Perelman M., Hewitt S.M., Cojacaru G., Goldberg I., Shahar I., Yaron P., Muler I., Campaner S., Amariglio N. (2004). Sil overexpression in lung cancer characterizes tumors with increased mitotic activity. Oncogene.

[14] Ouyang Y., Jin Y.B., Chen X.P., Zhang G.Y., Mao S.L., Ling F., Luo W. (2020). STIL is upregulated in nasopharyngeal carcinoma tissues and promotes nasopharyngeal carcinoma proliferation, migration and invasion. Neoplasma.

[15] Degoricija M., Korac-Prlic J., Vilovic K., Ivanisevic T., Haupt B., Palada V., Petkovic M., Karaman I., Terzic J. (2019). The dynamics of the inflammatory response during BBN-induced bladder carcinogenesis in mice. J. Transl. Med..

[16] Dobin A., Davis C.A., Schlesinger F., Drenkow J., Zaleski C., Jha S., Batut P., Chaisson M., Gingeras T.R. (2013). STAR: Ultrafast universal RNA-seq aligner. Bioinformatics.

[17] Liao Y., Smyth G.K., Shi W. (2014). featureCounts: An efficient general purpose program for assigning sequence reads to genomic features. Bioinformatics.

[18] Love M.I., Huber W., Anders S. (2014). Moderated estimation of fold change and dispersion for RNA-seq data with DESeq2. Genome Biol..

[19] Ito H., Tsunoda T., Riku M., Inaguma S., Inoko A., Murakami H., Ikeda H., Matsuda M., Kasai K. (2020). Indispensable role of STIL in the regulation of cancer cell motility through the lamellipodial accumulation of ARHGEF7-PAK1 complex. Oncogene.

[20] Izraeli S., Colaizzo-Anas T., Bertness V.L., Mani K., Aplan P.D., Kirsch I.R. (1997). Expression of the SIL gene is correlated with growth induction and cellular proliferation. Cell Growth Differ..

[21] Toyoshima H., Hunter T. (1994). p27, a novel inhibitor of G1 cyclin-Cdk protein kinase activity, is related to p21. Cell.

[22] Hume S., Dianov G.L., Ramadan K. (2020). A unified model for the G1/S cell cycle transition. Nucleic Acids Res..

[23] Youn M.J., Kim J.K., Park S.Y., Kim Y., Kim S.J., Lee J.S., Chai K.Y., Kim H.J., Cui M.X., So H.S. (2008). Chaga mushroom (Inonotus obliquus) induces G0/G1 arrest and apoptosis in human hepatoma HepG2 cells. World J. Gastroenterol..

[24] Wang Y., Zhou Y., Zhou H., Jia G., Liu J., Han B., Cheng Z., Jiang H., Pan S., Sun B. (2012). Pristimerin causes G1 arrest, induces apoptosis, and enhances the chemosensitivity to gemcitabine in pancreatic cancer cells. PLoS ONE.

[25] Zhao Y., Yu Y., Zhao W., You S., Feng M., Xie C., Chi X., Zhang Y., Wang X. (2019). As a downstream target of the AKT pathway, NPTX1 inhibits proliferation and promotes apoptosis in hepatocellular carcinoma. Biosci. Rep..

[26] Gupta A., Tsuchiya Y., Ohta M., Shiratsuchi G., Kitagawa D. (2017). NEK7 is required for G1 progression and procentriole formation. Mol. Biol. Cell..

[27] Erez A., Castiel A., Trakhtenbrot L., Perelman M., Rosenthal E., Goldstein I., Stettner N., Harmelin A., Eldar-Finkelman H., Campaner S. (2007). The SIL gene is essential for mitotic entry and survival of cancer cells. Cancer Res..

[28] Campaner S., Kaldis P., Izraeli S., Kirsch I.R. (2005). Sil phosphorylation in a Pin1 binding domain affects the duration of the spindle checkpoint. Mol. Cell. Biol..

[29] Li Y., Cheng X., Yan J., Jiang S. (2022). CTHRC1 facilitates bladder cancer cell proliferation and invasion through regulating the PI3K/Akt signaling pathway. Arch. Med. Sci..

[30] Yu X., Li S., Pang M., Du Y., Xu T., Bai T., Yang K., Hu J., Zhu S., Wang L. (2020). TSPAN7 Exerts Anti-Tumor Effects in Bladder Cancer Through the PTEN/PI3K/AKT Pathway. Front. Oncol..

[31] Liu H., Liu N., Zhao Y., Zhu X., Wang C., Liu Q., Gao C., Zhao X., Li J. (2019). Oncogenic USP22 supports gastric cancer growth and metastasis by activating c-Myc/NAMPT/SIRT1-dependent FOXO1 and YAP signaling. Aging (Albany NY).

[32] Borzi C., Calzolari L., Ferretti A.M., Caleca L., Pastorino U., Sozzi G., Fortunato O. (2019). c-Myc shuttled by tumour-derived extracellular vesicles promotes lung bronchial cell proliferation through miR-19b and miR-92a. Cell Death Dis..

[33] Fu Y., Su L., Cai M., Yao B., Xiao S., He Q., Xu L., Yang L., Zhao C., Wan T. (2019). Downregulation of CPA4 inhibits non small-cell lung cancer growth by suppressing the AKT/c-MYC pathway. Mol. Carcinog..

[34] Stine Z.E., Walton Z.E., Altman B.J., Hsieh A.L., Dang C.V. (2015). MYC, Metabolism, and Cancer. Cancer Discov..

[35] Bautista S.J., Boras I., Vissa A., Mecica N., Yip C.M., Kim P.K., Antonescu C.N. (2018). mTOR complex 1 controls the nuclear localization and function of glycogen synthase kinase 3beta. J. Biol. Chem..

[36] Wei C., Dong X., Lu H., Tong F., Chen L., Zhang R., Dong J., Hu Y., Wu G., Dong X. (2019). LPCAT1 promotes brain metastasis of lung adenocarcinoma by up-regulating PI3K/AKT/MYC pathway. J. Exp. Clin. Cancer Res..

[37] Jung M., Russell A.J., Liu B., George J., Liu P.Y., Liu T., DeFazio A., Bowtell D.D., Oberthuer A., London W.B. (2017). A Myc Activity Signature Predicts Poor Clinical Outcomes in Myc-Associated Cancers. Cancer Res..

[38] Zhang X.Y., Varthi M., Sykes S.M., Phillips C., Warzecha C., Zhu W., Wyce A., Thorne A.W., Berger S.L., McMahon S.B. (2008). The putative cancer stem cell marker USP22 is a subunit of the human SAGA complex required for activated transcription and cell-cycle progression. Mol. Cell.

[39] Penn L.J., Laufer E.M., Land H. (1990). C-MYC: Evidence for multiple regulatory functions. Semin. Cancer Biol..

[40] Facchini L.M., Penn L.Z. (1998). The molecular role of Myc in growth and transformation: Recent discoveries lead to new insights. FASEB J..

[41] Kim Y.H., Buchholz M.A., Chrest F.J., Nordin A.A. (1994). Up-regulation of c-myc induces the gene expression of the murine homologues of p34cdc2 and cyclin-dependent kinase-2 in T lymphocytes. J. Immunol..

[42] Haas K., Staller P., Geisen C., Bartek J., Eilers M., Moroy T. (1997). Mutual requirement of CDK4 and Myc in malignant transformation: Evidence for cyclin D1/CDK4 and p16INK4A as upstream regulators of Myc. Oncogene.

