# Supplementary material for: STIL Promotes Tumorigenesis of Bladder Cancer by Activating PI3K/AKT/mTOR Signaling Pathway and Targeting C-Myc
Source: Cancers (Basel). 2022 Nov 24;14(23):5777. doi: 10.3390/cancers14235777 (PMC9739707; doi:10.3390/cancers14235777)
Supplement: Supplementary file 1 [file cancers-14-05777-s001.zip › FigureS1-4ú║Supplementary figure.pdf]

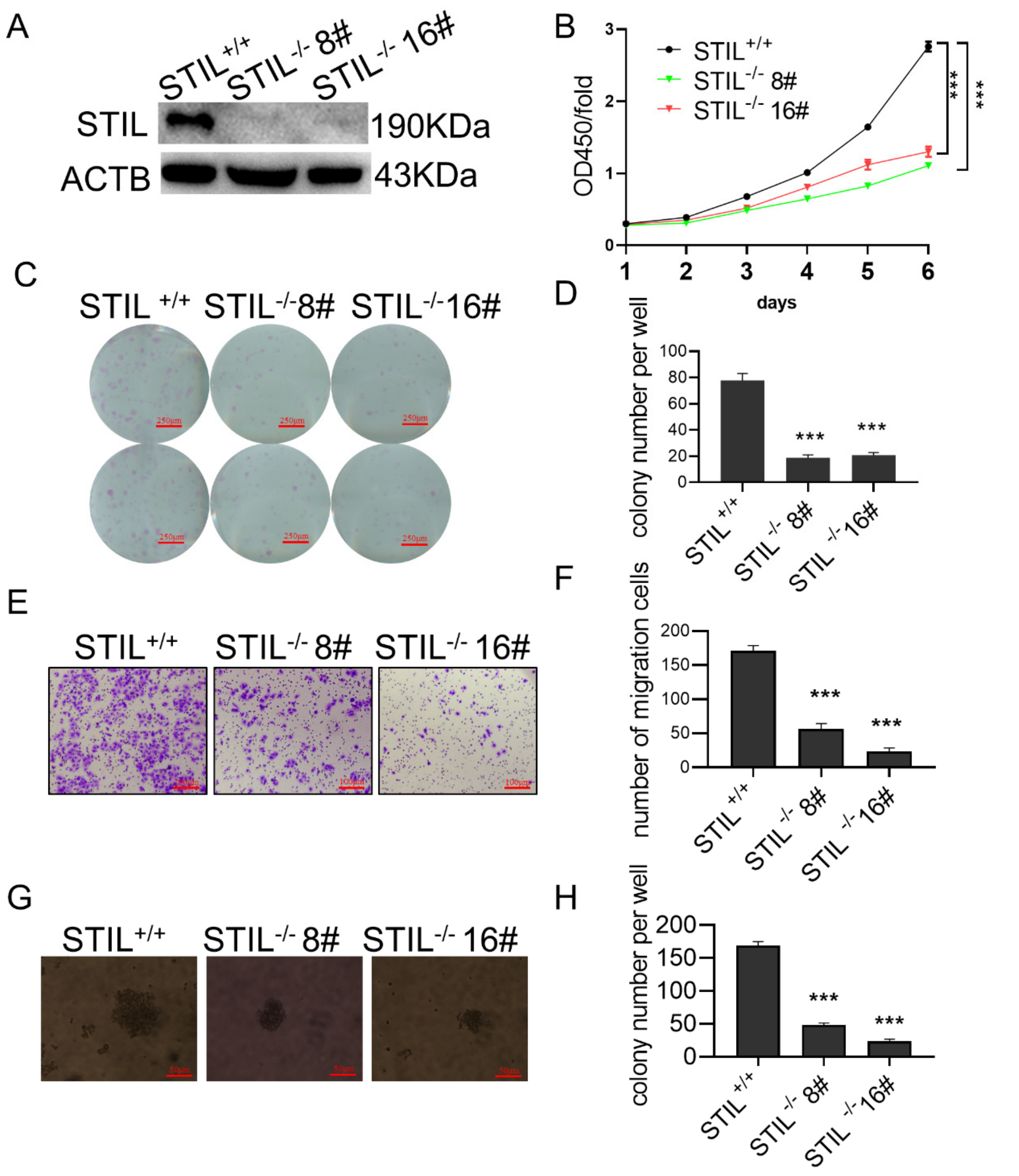

Figure S1. In the T24 cell line, knockout of STIL inhibited BC tumorigenesis in vitro. **(A)** After screening by western blot, the exact STIL-knockout cells ($STIL^{-/-}$8# and $STIL^{-/-}$16#) were obtained. **(B)** CCK-8 assay. **(C)** Representative microscope images and **(D)** quantitative analysis of colony formation assays. **(E)** Representative microscope images and **(F)** quantitative analysis of transwell migration assay. **(G)** Representative microscope images and **(H)** quantitative analysis of soft agar assay. All experiments were conducted with the following groups: control group ($STIL^{+/+}$ cell) and experimental groups ($STIL^{-/-}$ 8# cell and $STIL^{-/-}$16# cell). (* $P < 0.05$, ** $P < 0.01$, and *** $P < 0.001$.)

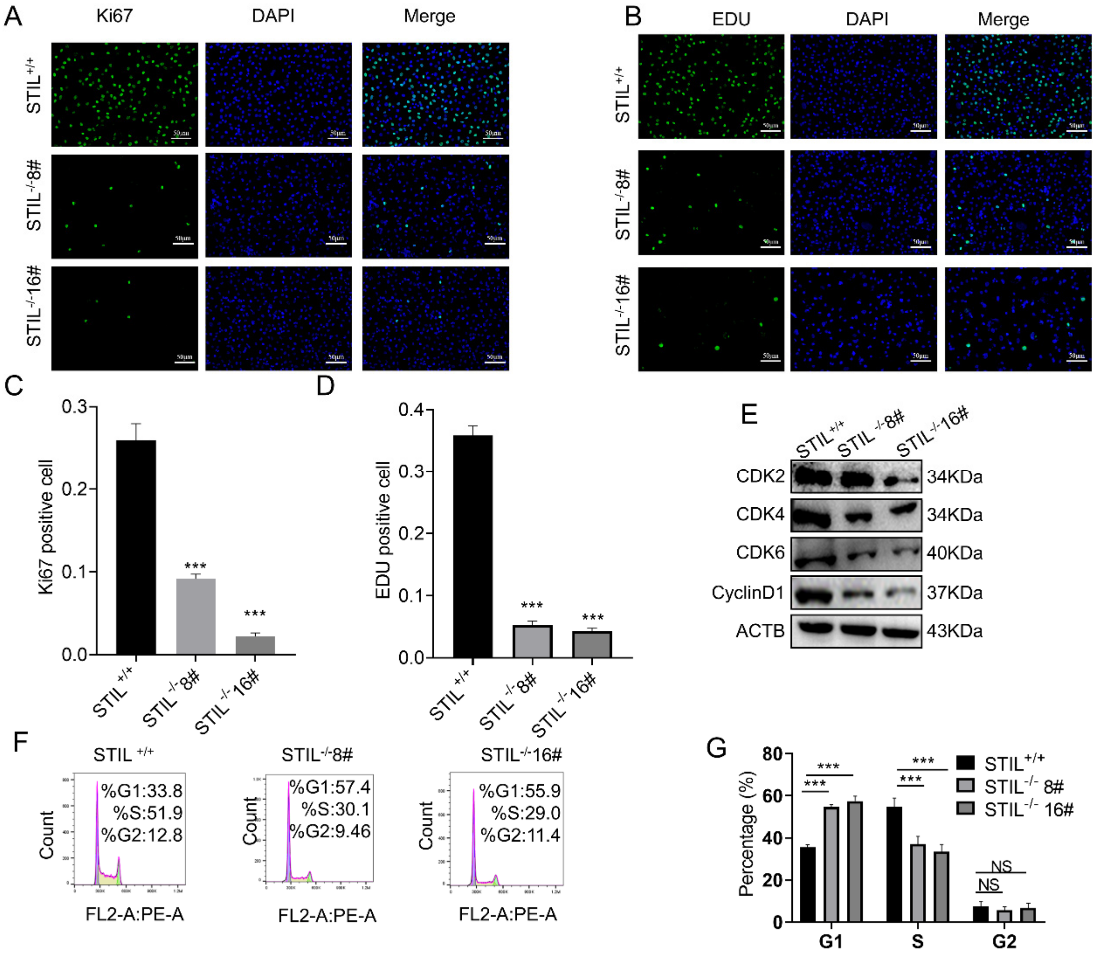

Figure S2. In the T24 cell line, STIL played a vital role in the proliferation and cell cycle of BC**.** Representative microscope images of (**A**) Ki67 staining and (**B**) EDU staining. The green color represents Ki67 and EDU, respectively, and the blue color represents DAPI. The quantitative analysis of (**C**) Ki67 staining and (**D**) EDU staining. **(E)** Representative images of western blotting of cyclin D1 and cell-cycle-related proteins CDK2/4/6. **(F)** Representative images of flow cytometry for propidium iodide (PI) staining and (**G**) quantitative analysis of cell cycle phase**.** EDU, 5-ethynyl-2-deoxyuridine. (* $P < 0.05$, ** $P < 0.01$, and *** $P < 0.001$.)

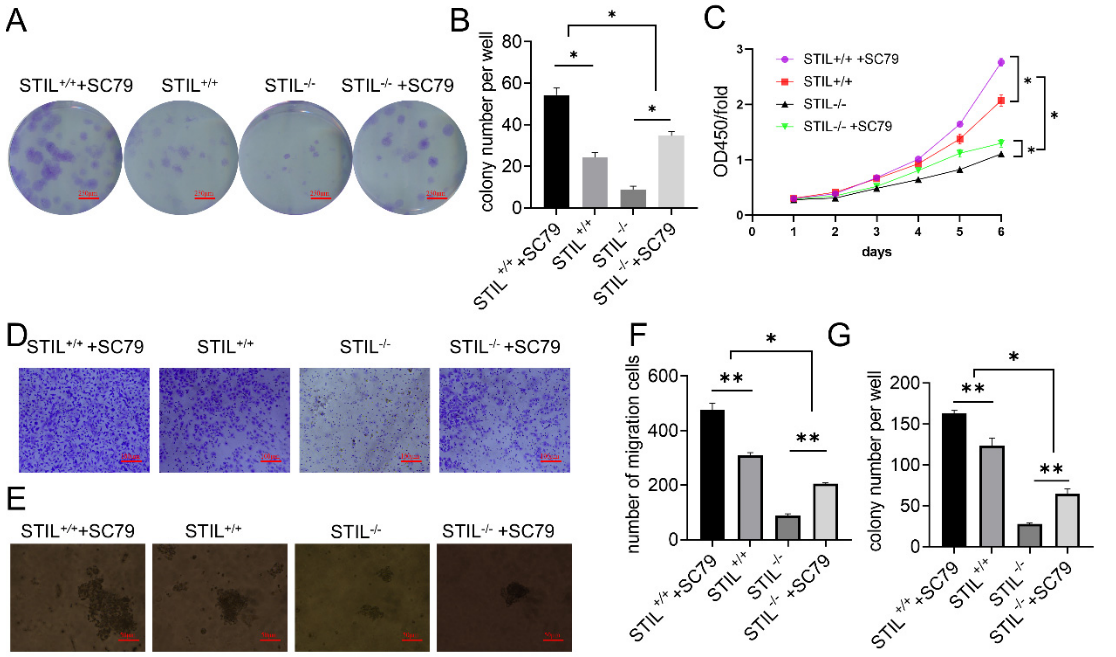

Figure S3. In the T24 cell line, SC79 treatment partially reversed the effect of STIL knockout on cell tumorigenesis. **(A)** Representative microscope images and **(B)** quantitative analysis of colony formation assays. **(C)** Cell proliferation curves of CCK-8 assays. **(D)** Representative microscope images and **(F)** quantitative analysis of transwell migration assay. **(E)** Representative microscope images and **(G)** quantitative analysis of soft agar assay. In all the above experiments, four groups of cells were tested simultaneously ($STIL^{+/+}$+SC79, $STIL^{+/+,}$ $STIL^{-/-}$, $STIL^{-/-}$+SC79). (* $P < 0.05$, ** $P < 0.01$, and *** $P < 0.001$.)

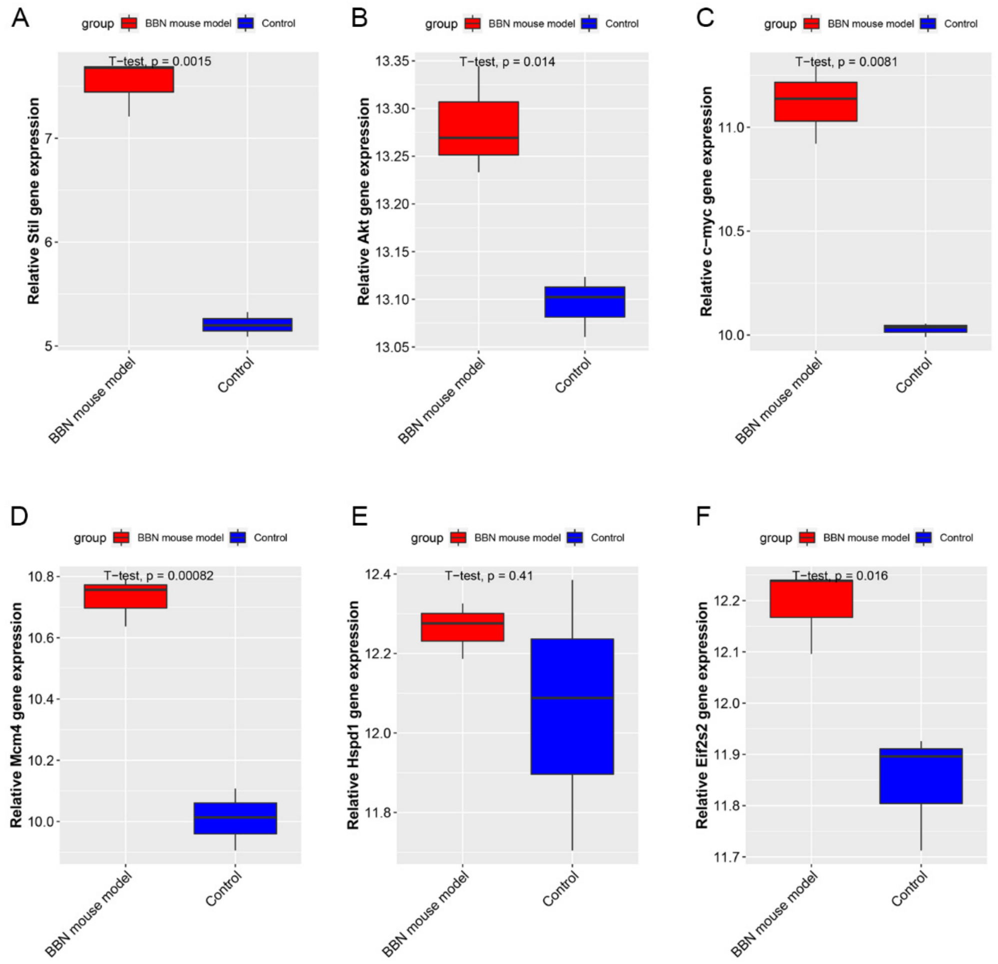

Figure S4. STIL and some other genes mRNA expression was significantly upregulated in BBN induced bladder cancer tissues, compared with control samples. **(A)** STIL **(B)** AKT **(C)** c-myc **(D)** MCM4 **(E)** HSPD1 **(F)** EIFIS2.
