# Supplementary material for: STIL Promotes Tumorigenesis of Bladder Cancer by Activating PI3K/AKT/mTOR Signaling Pathway and Targeting C-Myc
Source: Cancers (Basel). 2022 Nov 24;14(23):5777. doi: 10.3390/cancers14235777 (PMC9739707; doi:10.3390/cancers14235777)

Original pictures of western blot bands in Figure 2D:

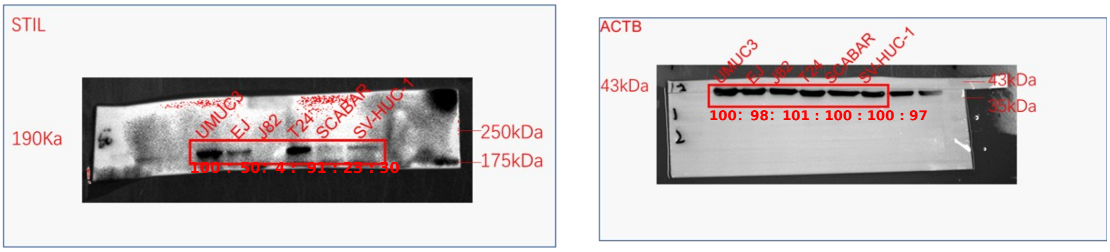

Original pictures of western blot bands in Figure 3A:

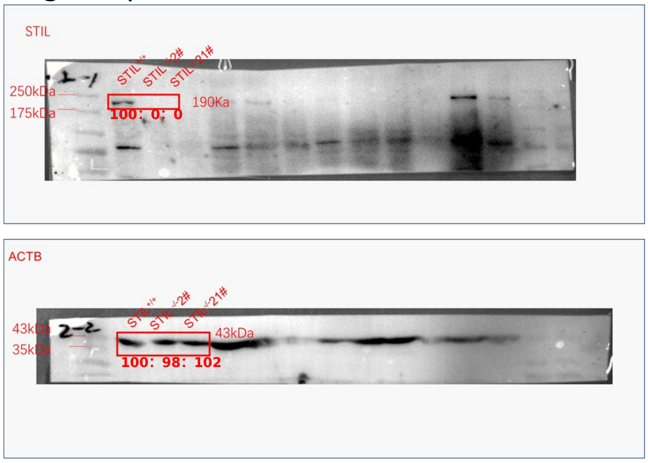

Original pictures of western blot bands in Figure4A:

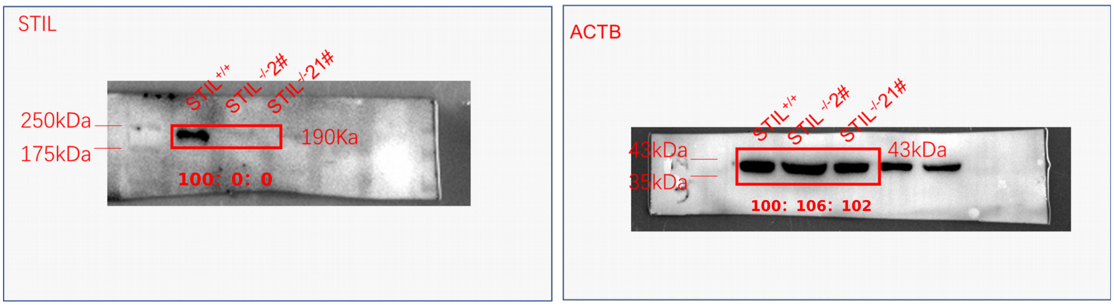

Original pictures of western blot bands in Figure5G:

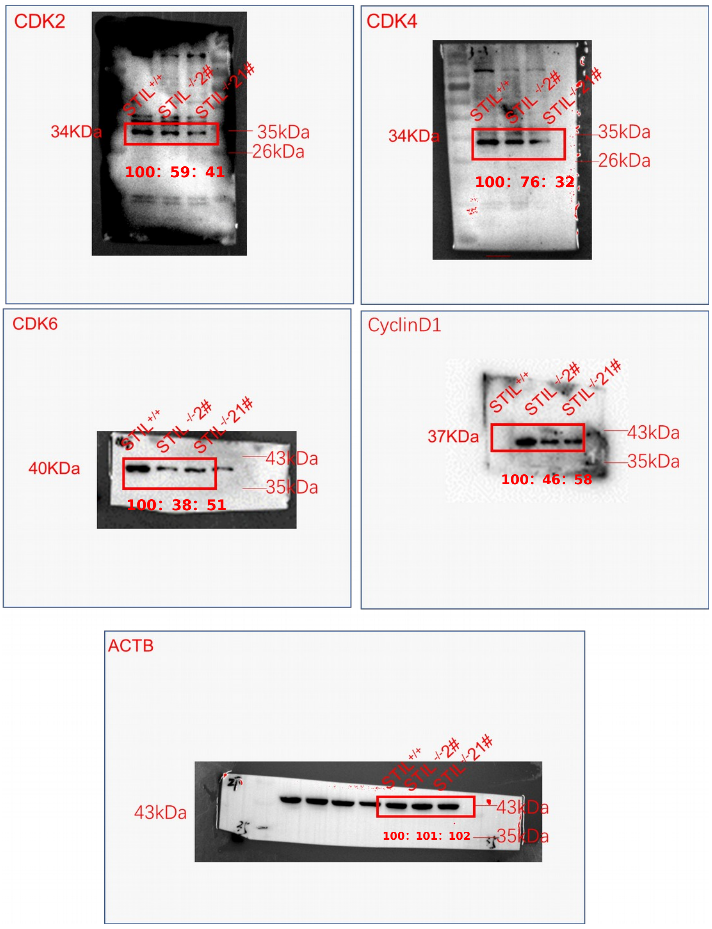

Original pictures of western blot bands in Figure6E:

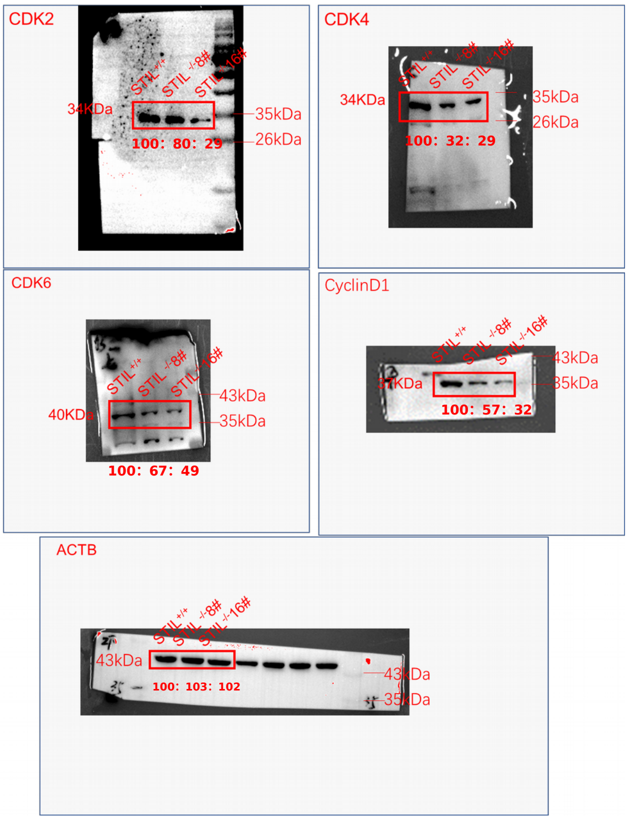

Original pictures of western blot bands in Figure 8H:

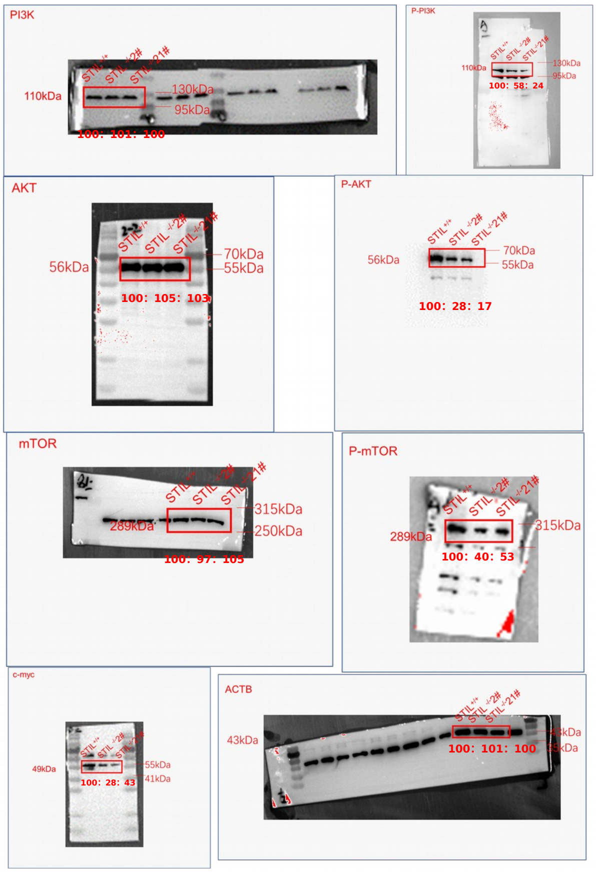

Original pictures of western blot bands in Figure 8I:

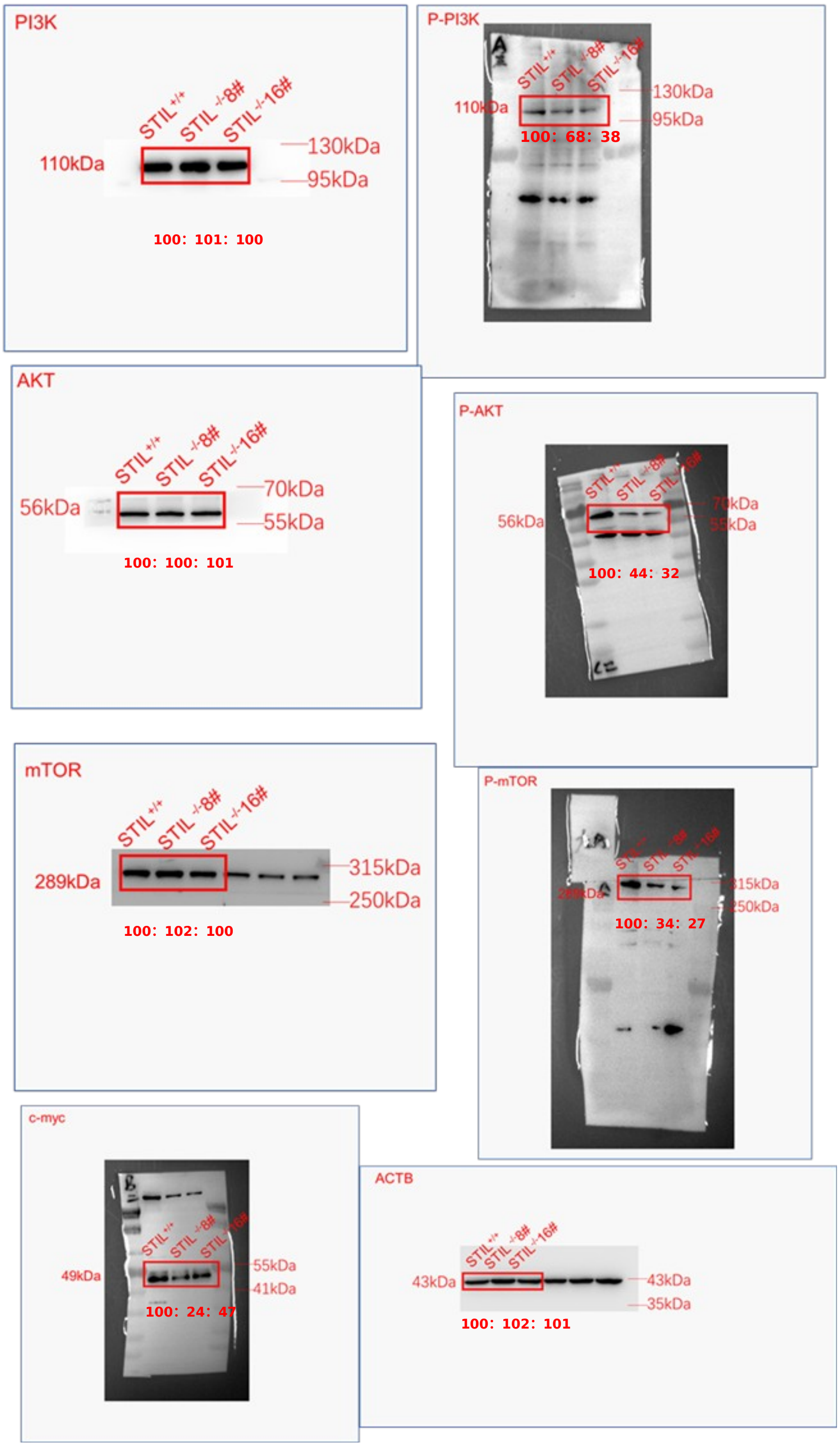

Supplement: Supplementary file 1 [file cancers-14-05777-s001.zip › File S1ú║Original pictures of western blot.pdf]
