# Supplementary material for: STIL Promotes Tumorigenesis of Bladder Cancer by Activating PI3K/AKT/mTOR Signaling Pathway and Targeting C-Myc
Source: Cancers (Basel). 2022 Nov 24;14(23):5777. doi: 10.3390/cancers14235777 (PMC9739707; doi:10.3390/cancers14235777)
Supplement: Supplementary file 1 [file cancers-14-05777-s001.zip › Table S1ú║Real-Time quantitative PCR Primer sequences.pdf]

Table S1: Real-Time quantitative PCR Primer sequences.

Real-Time quantitative PCR Primer sequences		
STIL	Forward primer:	CCCAACGCCAACTGGAGATTT
	Reverse primer:	AGTCGGATGGTCTTCTCAGTC
ABCE1	Forward primer:	GGAATGCAAAAAGAGTTGTCCTG
	Reverse primer:	CGAGGGATAGGCAACCTGTG
DDX18	Forward primer:	ATGTCACACCTGCCGATGAAA
	Reverse primer:	CCCTGAAACTTTAGGTTCCGC
VRK1	Forward primer:	CCTCGTGTAAAAGCAGCTCAA
	Reverse primer:	GCCAATGGGTAATCCTACTTTCC
CBX3	Forward primer:	TAGATCGACGTGTAGTGAATGGG
	Reverse primer:	TGTCTGTGGCACCAATTATTCTT
HSPD1	Forward primer:	ATGCTTCGGTTACCCACAGTC
	Reverse primer:	AGCCCGAGTGAGATGAGGAG
MCM4	Forward primer:	GACGTAGAGGCGAGGATTCC
	Reverse primer:	GCTGGGAGTGCCGTATGTC
DDX21	Forward primer:	GAGGAGCCATCTCAAAATGACA
	Reverse primer:	GGGTTACAGTCCGGTTCAGG
EIF2S2	Forward primer:	AAGGGGATACCCAAACAGAGG
	Reverse primer:	TTCATCAGCTTCCAAATCCTTGT
ACTB	Forward primer:	AGAGCTACGAGCTGCCTGAC
	Reverse primer:	AGCACTGTGTTGGCGTACAG
